# Treadmill Exercise Reshapes Cortical Astrocytic and Neuronal Activity to Improve Motor Learning Deficits Under Chronic Alcohol Exposure

**DOI:** 10.1007/s12264-024-01226-x

**Published:** 2024-05-28

**Authors:** Linglin Liu, Lanzhi Luo, Ji-an Wei, Xintong Xu, Kwok-Fai So, Li Zhang

**Affiliations:** 1 Key Laboratory of CNS Regeneration (Ministry of Education), Guangdong-Hong Kong-Macau Institute of CNS Regeneration, Jinan University, Guangzhou, 510632 China; 2 College of Life Science and Technology, Jinan University, Guangzhou, 510632 China; 3 State Key Laboratory of Brain and Cognitive Science, Li Ka Shing Faculty of Medicine, The University of Hong Kong, Pok Fu Lam, Hong Kong SAR China; 4 Neuroscience and Neurorehabilitation Institute, University of Health and Rehabilitation Sciences, Qingdao, 266113 China; 5 Center for Exercise and Brain Science, School of Psychology, Shanghai University of Sport, Shanghai, 200438 China; 6 The First Affiliated Hospital of Xinxiang Medical University, Xinxiang, 453003 China

**Keywords:** Alcohol abuse, Motor learning, Dendritic spine, Reactive astrocyte

## Abstract

Alcohol abuse induces various neurological disorders including motor learning deficits, possibly by affecting neuronal and astrocytic activity. Physical exercise is one effective approach to remediate synaptic loss and motor deficits as shown by our previous works. In this study, we unrevealed the role of exercise training in the recovery of cortical neuronal and astrocytic functions. Using a chronic alcohol injection mouse model, we found the hyperreactivity of astrocytes along with dendritic spine loss plus lower neuronal activity in the primary motor cortex. Persistent treadmill exercise training, on the other hand, improved neural spine formation and inhibited reactive astrocytes, alleviating motor learning deficits induced by alcohol exposure. These data collectively support the potency of endurance exercise in the rehabilitation of motor functions under alcohol abuse.

**Supplementary Information:**

The online version contains supplementary material available at 10.1007/s12264-024-01226-x.

## Introduction

Alcohol abuse is a worldwide health issue that affects more than 8% of the male population [1] and may cause various mental disorders and cognitive deficits. Within the brain, persistent alcohol exposure dramatically changed the homeostasis of neurons and synapses, as supported by cortical and subcortical neuronal degenerations in patients [2]. Using animal models, people have identified aberrant synaptic pruning and loss of excitatory synapses in the medial prefrontal cortex (mPFC) [3]. Besides neurons, glial cells were found to be prominently affected by alcohol abuse, such as the change of signaling transduction in microglia [4] and compromised oligodendrocyte cell lineage differentiation [5]. As the major metabolic center for the brain, astrocytes possess aldehyde dehydrogenase for the intoxication of alcohol [6]. Their morphological and functional switches during alcohol exposure thus have been investigated. In human patients, alcohol abuse leads to astrocyte swelling, higher reactivity, and proliferation [2]. Rodent models of alcohol exposure also showed morphological [7] and transcriptomic changes [8] in astrocytes. All these studies showed alcohol-dependent changes in neurons and astrocytes but lacked sufficient *in vivo* evidence for their cellular activity.

Exercise training is one effective non-drug intervention for psychiatric and neurological disorders. Our group previously demonstrated the effectiveness of aerobic exercise in alleviating anxiety-like behaviors induced by environmental stress [9–11]. Moreover, persistent treadmill exercise recovered motor learning deficits in a mouse model of cocaine exposure, *via* potentiating cortical synaptogenesis and neural network activity [12]. Other groups have also reported the amelioration of alcohol abuse-induced neural deficits by exercise training, including mental disorders and cognitive deficits [13]. Few studies, however, explored the intervention of alcohol-induced motor deficits by exercise training. Since deficits in complex motor learning are one of the core symptoms of chronic alcohol intake [14], which can induce prominent synaptic loss [15], we thus explored if endurance exercise improved alcohol-dependent motor learning deficits, and investigated the possible involvement of synaptic plasticity. Moreover, as astrocyte-neuron crosstalk is crucial for maintaining homeostasis of neural synapse [16, 17], the study of astrocyte morphology and activity will be of interest to understanding the interplay between neurons and glial cells during exercise intervention.

By establishing a chronic alcohol administration model in adult mice, we demonstrated the effectiveness of 18-day treadmill exercise in relieving motor learning deficits induced by alcohol exposure. Further *in vivo* imaging assays showed the restoration of normal spine formation, plus the modulation of neuronal and astrocytic calcium activity by exercise training in alcohol-exposed mice. Manipulation of astrocytic activity demonstrated the direct relationship between astrocytes and motor learning. Our results thus provide some previously unrecognized mechanisms of neuron-astrocyte crosstalk in exercise intervention.

## Materials and Methods

### Experimental Animals

Male C57BL/6J mice (5–6 weeks old) were acquired from the Guangdong Medical Laboratory Animal Center. Male Thy1-YFP mice (5 weeks old) were obtained from the Jackson Laboratory and bred in-house. All animals were housed in a standard animal facility under a 12-h normal light/dark cycle (lights on at 08:00, off at 20:00) with *ad libitum* access to food and water. Ethical approval for all animal experimental protocols has been obtained from the Ethics Committee of Experimental Animals at Jinan University, following IACUC guidelines for animal research. The sample size was determined based on current literature within the same field, considering animal welfare requirements and ethical codes. While no systematic randomization method was employed, animals were randomly assigned to respective groups.

To establish a model of chronic alcohol exposure, ethanol (1 g/kg daily) or vehicle (0.9 % saline of equal volume) was administered intraperitoneal (IP) for 18 consecutive days. The ethanol injection was performed at 9 pm each day. In the exercise intervention model (Alcohol+Ex), mice were also enrolled in the treadmill training (JD-PT Model, Jide Instrument, Shanghai, China) at 9 am each day (i.e. 12 h after the alcohol injection). The exercise training also lasted for 18 days. The running velocity was set at 8 m/min during the first 3 days and was elevated to a moderate-to-high intensity (10–12 m/min, 1 h daily) in the remaining 15 days. The sedentary (no exercised) animals were placed on the apparatus with the fixed treadmill for 1 h as a control.

### Stereotaxic Injection of Viral Vectors

Mice were anesthetized with isoflurane. After hair shaving, local sterilization, and incision of head skins, the primary motor cortex (AP: +1.2 mm; ML: ±2.0 mm; DV: –0.60 mm) was localized under the stereotaxic instrument (RWD, China). Viral vectors were injected using a glass micropipette connected to a microinjection pump (Nanoliter 2010, WPI, USA), at an angle of 60° to avoid extra damage to the relevant image, the injection hole was made at AP: +2.0 mm by the high-speed micro-drill (OmniDrill35, WPI, USA), and viral vectors were delivered into the target region at speed of 50 nL/min. When the injection was finished, the injection needle was retained for 10 min before withdrawal. All viral vectors are listed in Table S1.

### qRT–PCR

The whole brain was extracted, and the M1 was carefully dissected under a brain mold and grinded in TRIzol (Ambion, USA). Then the lysate was purified with an EZ-10 Total RNA Mini-Preps Kit (Sangon, China) to collect RNA, which was stored at –80 ℃. Before reverse transcription, the concentration of RNA was measured by NanoDrop (Thermo Fisher, USA). A total RNA of 200 ng was used for *in vitro* reverse transcription by PrimeScript RT Reagent Kit (TaKaRa, Japan). The cDNA was then analyzed by qRT–PCR *via* TB GreenPremix Ex Taq (TaKaRa, Japan) in CFX96 Dx System (Bio-Rad, USA). The RNA abundance of each gene was normalized to a housekeeping gene *Gapdh*. The following primers were employed in this experiment: (1) *Gapdh*, F: 5’-AGGTC GGTGT GAACG GATTT G-3’, R: 5’-TGTAG ACCAT GTAGT TGAGG TCA-3’. (2) *Slc29a1*, F: 5’-CAGCC TCAGG ACAGG TATAA GG-3’, R: 5’-GTTTG TGAAA TACTT GGTTG CGG-3’. (3) *P2rx4*, F: 5’-CTGGT GTGCC AACGA GGAAT A-3’, R: 5’-AGACG GAATA TGGGG CAGAA G-3’. (4) *P2ry12*, F: 5’-ATGGA TATGC CTGGT GTCAA CA-3’, R: 5’-AGCAA TGGGA AGAGA ACCTG G-3’. (5) *Slc6a11*, F: 5’-TGTTG AGCGT AGCTG GAGAG A-3’, R: 5’-AGCAG ATGAA AAACA CCACG TA-3’. (6) *Slc1a3*, F: 5’-ACCAA AAGCA ACGGA GAAGA G-3’, R: 5’-GGCAT TCCGA AACAG GTAAC TC-3’. (7) *Aldh1l1*, F: 5’- CAGGA GGTTT ACTGC CAGCT A-3’, R: 5’- CACGT TGAGT TCTGC ACCCA-3’.

### Immunofluorescent Staining

Mice were deeply anesthetized and subjected to cardiac perfusion using 0.9% PBS. The brain tissue was dissected and fixed within a 4% paraformaldehyde (PFA) solution overnight and was transferred to a 30% sucrose solution for 1 day dehydration. The M1 region of the brain was sliced into 40 μm coronal sections by a sliding microtome (SM2010R, Leica, Germany). The brain slices were washed with PBS and incubated in a blocking solution at room temperature for 2 h. After blocking, the brain slices were incubated with diluted primary antibodies (1:1000 or 1:500) and incubated at 4℃ for 36 h. The slices were then washed with PBS buffer to remove unbound primary antibodies. Subsequently, the secondary antibodies (diluted to 1:500) were added and incubated at room temperature in the dark for 2 h. Afterward, the brain sections were washed again, mounted on slides, and air-dried at room temperature. After mounting the coverslip, the slides were imaged using a laser confocal microscope (LSM700, Zeiss, Germany). All antibodies used are listed in Table S1.

### Behavioral Assays

#### Open Field

The open field test was conducted to assess the locomotor activity and emotional state of mice. The test mouse was placed in a square test arena (50 cm × 50 cm × 40 cm) for free exploration within 5 min. EthoVision 8.0 software (Noldus, Wageningen, The Netherlands) was used to record and analyze the total distance traveled and the duration of time spent in the center area.

#### Elevated Plus-maze

The elevated plus-maze test was conducted to evaluate the anxiety-like behavior of mice. The maze consisted of two open arms (30 cm × 5 cm), two closed arms (30 cm × 5 cm), and a central platform (5 cm × 5 cm). The mouse was allowed to freely explore the maze for 5 min, and its movements were recorded using a camera. The movement path was analyzed using EthoVision 8.0 software to determine the duration of time spent in the open arms.

#### Accelerating Rotarod

The mouse was acclimated to the rotarod apparatus (Ugo Basile, Italy) at a speed of 2 r/min. Subsequently, it underwent an acceleration of 5 rad/min^2^, commencing from 2 r/min to 80 r/min. The latency to fall was recorded and the test was repeated over three consecutive days.

#### Pole Climbing

A vertical pole with a diameter of 2 cm and a height of 50 cm was used. The mouse was first enrolled in an adaptation session before the test. The time taken to descend from the top of the pole to the platform was recorded for 3 consecutive days.

#### Beam Walking

A horizontally placed wooden beam (12 mm diameter) was placed at 1 m above the ground. At the end of the beam, a black box was designated as the destination. The duration taken by the mouse from the starting point to the escape box was recorded over three consecutive days.

#### Grip Strength

The grip test is used to evaluate the muscle strength of the four limbs. Mice were placed on a grid with their fore/hind paws (4 paws) onto a grid and were gently pulled backward until they released their grip. A grip strength meter (RWD, China) attached to a force transducer measured the peak force generated. Results from three trials were completed and averaged.

### Chemogenetic Manipulation

The rAAV-GfaABC1D-hM4D(Gi)-mCherry, or rAAV-GfaABC1D-GFP was injected into M1 as previously described. After one week of recovery, the 18-day alcohol infusion was initiated (1 g/kg daily), and 10 mg/kg clozapine-N-oxide (CNO) or 0.9% saline was injected every other day.

### Two-Photon Calcium Imaging

The calcium activities of M1 neurons or astrocytes were recorded by transcranial 2-photon imaging. GCaMP6s was used for neuronal imaging, while GCaMP6f was adopted for astrocyte imaging, mainly due to the different kinetics between neuronal and astrocytic calcium spikes [18]. After expressing GCaMP6s or GCaMP6f within a specific cell type, the skull above M1 was removed, and a circular glass coverslip (Vetbond Tissue Adhesive, 3M, USA) was placed. An LSM780 two-photon microscopy (Zeiss, Germany) was used to record the calcium activities of astrocytes and neurons. Calcium activities were recorded from layer V somas (500–600 μm depth) at 2 Hz with a water-immersed objective (20×, 1.1 NA, Zeiss, Germany). Imaging utilized a 920 nm excitation laser. Image collection and analysis were performed using ImageJ with Fiji plug-in (NIH, Bethesda, USA). Normalized *F* values were obtained by calculating ∆*F*/*F*min (∆*F* = *F* – *F*_min_), where Fmin is defined as the minimum value of all fluorescence readings during the recording period. After normalizing, the summation of all ∆F/Fmin values was employed as the total integrated Ca^2+^. When the recorded ∆F/Fmin exceeds three times the baseline level, a calcium peak was defined.

### Dendritic Spine Imaging

The apical dendritic spine of M1 pyramidal neurons from Thy1-YFP mice was described using two-photon microscopy, following established approaches in our laboratory [12, 19]. Briefly speaking, the skull above the motor cortex was thinned, and dendritic spine images were captured with LSM780 two-photon microscopy, using a 20× water-immersion objective. The z-stacked image series (thickness: 80–120 μm; layer interval: 1.1 μm) was obtained for analysis using Image J software. Around 100–150 dendritic spines per group were analyzed for quantification. A stable spine was defined as one that appeared in both imaging sessions. An eliminated spine was observed in the first image but absent in the second. A newly generated spine was absent in the first image but present in the second. To analyze spine density, dendritic shafts containing 8–15 spines were selected, and the density was calculated as spine number per dendritic length (in μm).

### Morphological Analysis of Astrocytes

The morphology of individual astrocytes was obtained using confocal microscopy (40× objective). Neurolucida 360 package was used to extract the complete cell morphology, followed by Sholl analysis. The soma size, dendritic length, and branch count were calculated.

### Statistical Analysis

GraphPad Prism 9.0 software was used for data analysis and figure plotting. All data were presented as mean ± standard error of means (SEM). A normality test was first applied. Data fitting the normal distribution was compared using unpaired *t*-tests or one-way analysis of variance (ANOVA). For the analysis involving 2 factors (such as the accelerating rotarod), two-way ANOVA was employed. For data with nonparametric distribution, Kruskal-Wallis was applied. A statistical significance was considered at *P* < 0.05.

## Results

### Physical Exercise Relieves Motor Learning Deficits Under Alcohol Abuse

We first characterized the behavioral phenotype of mice with chronic alcohol infusion. By intraperitoneal injection of 10% alcohol for 18 consecutive days, we evaluated the behavioral phenotype of male mice (Fig. S1A). In general, these alcohol-infused mice had intact anxiety-like behaviors or general locomotor ability (Fig. S1B–E). When examining the acquisition for complex motor tasks, however, alcohol administration induced deterioration of motor performance in the accelerating rotarod test, polo climbing, and beam walking tasks (Fig. S1F–I).

**Fig. 1:**
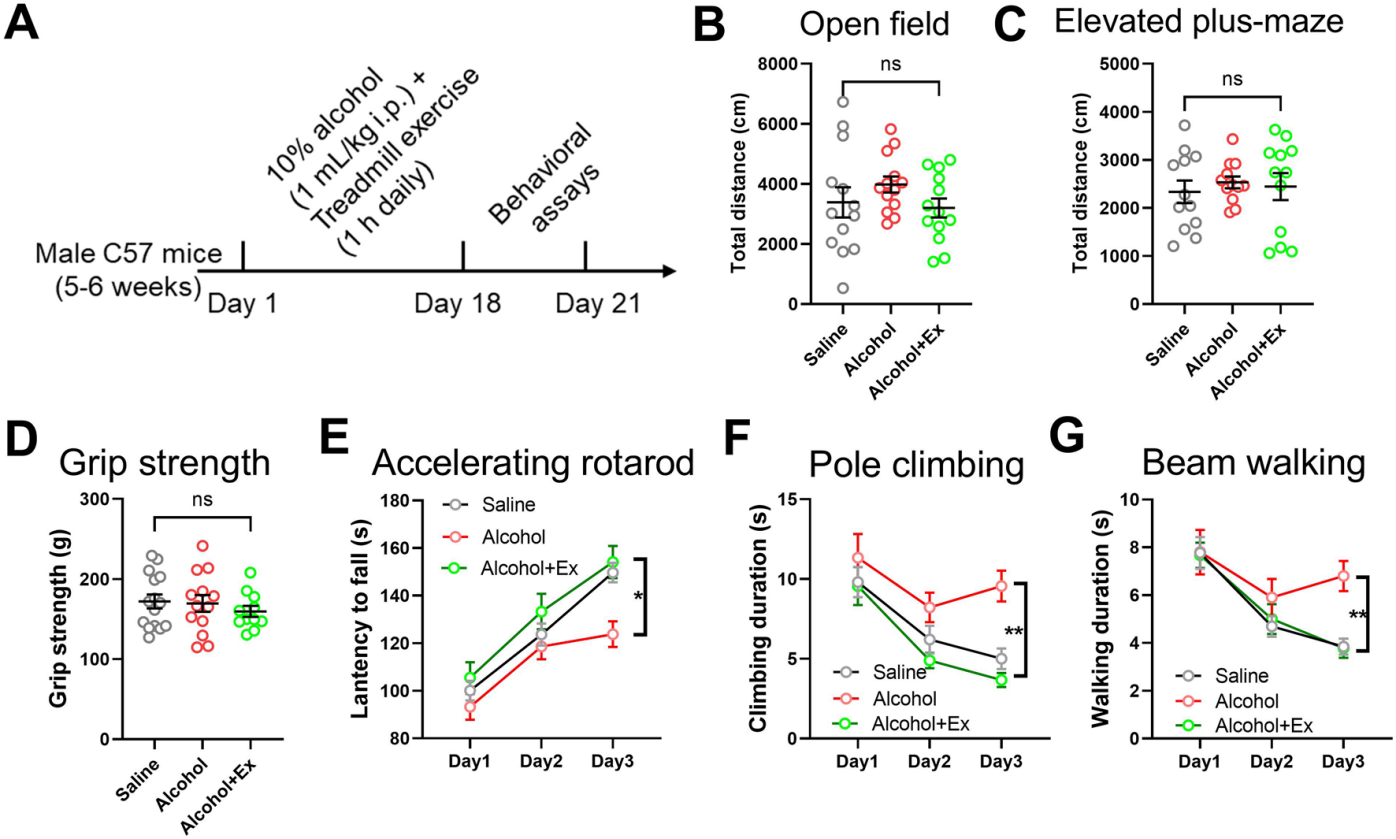
Treadmill exercise alleviates alcohol-induced motor learning deficits. **A** Experimental scheme. **B** Total distance in the open field. One-way ANOVA, *F*_(2, 36)_ = 1.183, *P* = 0.3179. **C** Total distance traveled in the elevated plus-maze. One-way ANOVA, *F*_(2, 36)_ = 0.1963, *P* = 0.8227. **D** Hindlimb grip strength. One-way ANOVA, *F*_(2, 36)_ = 0.4797, *P* = 0.6229. **E** Latency to fall during the 3-day accelerating rotarod assay. Two-way ANOVA for the group factor, *F*_(2, 47)_ = 4.544, *P* = 0.0157. **F** Time spent climbing the vertical pole. Two-way ANOVA for the group factor, *F*_(2, 47)_ = 6.979, *P* = 0.0039. **G** Duration in accomplishing the horizontal walking beam. Two-way ANOVA for the group factor, *F*_(2, 47)_ = 5.939, *P* = 0.0077. *n* = 13 mice per group in **(B**–**G)**. ns, no significant difference; *P < 0.05; **P < 0.01. All data were presented as mean±SEM.

We next examined the amelioration effect of exercise, based on our established models of exercise in improving motor learning functions in both naïve and cocaine-exposed mice [12, 19]. By combining the above chronic alcohol injection model with 18-day treadmill exercise (10–12 m/min, 1 h daily) (Fig. 1A), mice presented normal locomotor activity (Fig. 1B–C) or normal grip strength (Fig. 1D). When examining the acquisition for complex motor tasks, however, treadmill exercise attenuated the impairments in the accelerating rotarod test (Fig. 1E). Moreover, during the pole climbing or beam walking task, exercise also improved the overall performance of alcohol-infused mice (Fig. 1F–G). These results demonstrated the impaired motor learning function under alcohol abuse, and relief of these behavioral deficits by exercise intervention.

### Exercise Reshapes Synaptic Structure and Functions in Motor Cortex

**Fig. 2:**
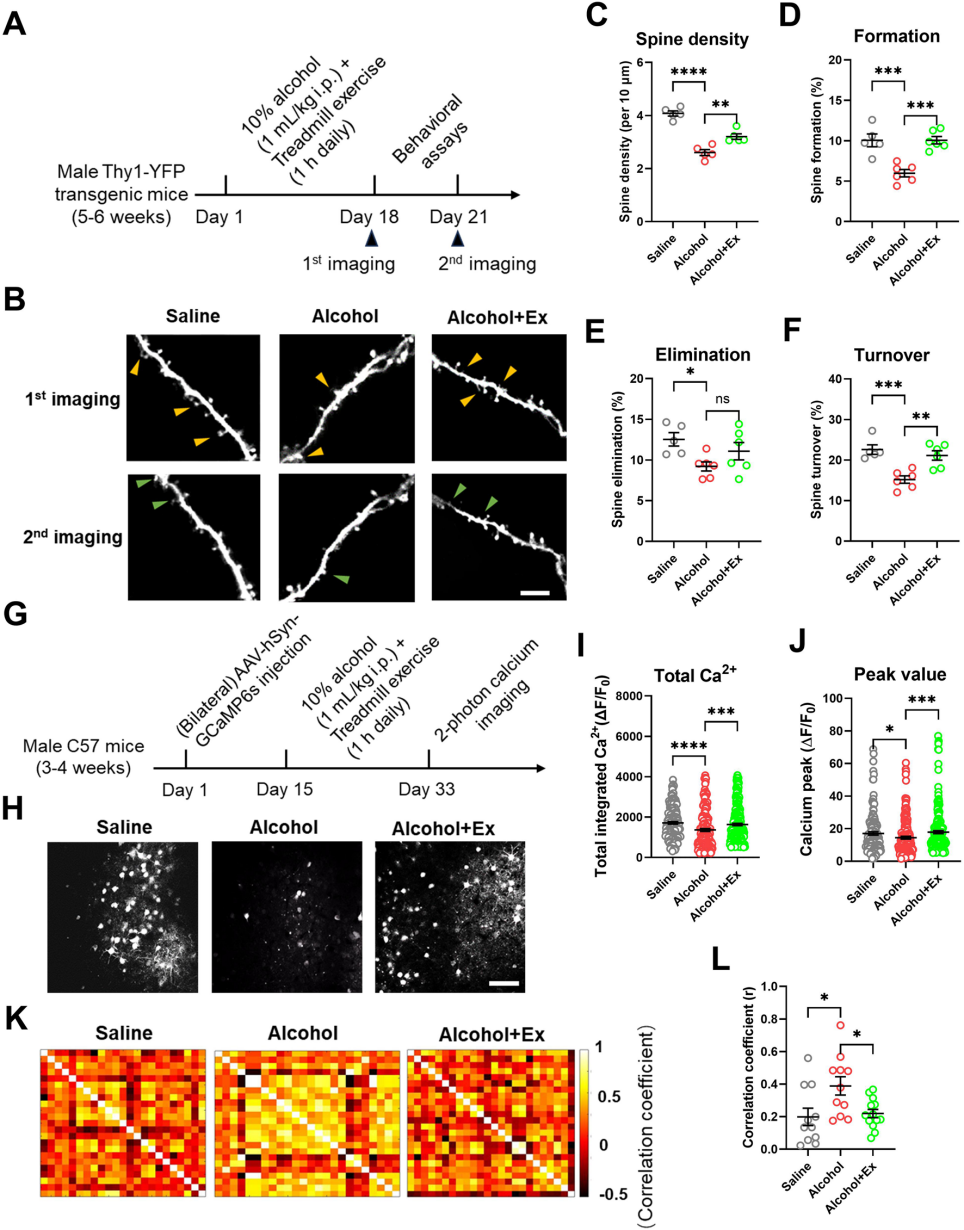
Exercise training restores structural and functional homeostasis of cortical neurons. **A** Experimental scheme of 2-photon *in vivo* spine imaging. **B** Sample images of apical spines of M1 pyramidal neurons across 2 imaging sessions. Scale bar, 5 μm. **C** Density of apical spines. One-way ANOVA, *F*_(2, 14)_ = 50.29, *P* < 0.0001. Tukey’s *post-hoc* comparison, Saline *vs* Alcohol, *P* < 0.0001; Alcohol *vs* Alcohol+Ex, *P* = 0.0040. **D** Formation rate of spines between 1^st^ and 2^nd^ imaging session. One-way ANOVA, *F*_(2, 14)_ = 17.96, *P* = 0.0001. Tukey’s *post-hoc* comparison, Saline *vs* Alcohol, *P* = 0.0005; Alcohol *vs* Alcohol+Ex, *P*=0.0003. **E** Elimination rate of dendritic spines. One-way ANOVA, *F*_(2, 14)_ = 3.764, *P* = 0.0492. Tukey’s *post-hoc* comparison, Saline *vs* Alcohol, *P*=0.0412; Alcohol *vs* Alcohol+Ex, *P* = 0.2776. **F** Overall turnover rate of spines. One-way ANOVA, *F*_(2, 14)_ = 13.00, *P*=0.0006. Tukey’s *post-hoc* comparison, Saline *vs* Alcohol, *P* = 0.0009; Alcohol *vs* Alcohol+Ex, *P* = 0.0037. *n* = 5, 6, and 6 mice in the Saline, Alcohol, and Alcohol+Ex group, respectively, in (**C**–**F**). **G** Experimental scheme of neuronal calcium imaging. **H** Time-series stacking images showing the fluorescence of GCaMP6s within M1 over the imaging time window. Scale bar, 150 μm. **I** Total integrated calcium strength. Nonparametric Kruskal-Wallis test statistic = 25.94, *P* < 0.0001. Dunn’s *post-hoc* comparison, Saline *vs* Alcohol, *P* < 0.0001; Alcohol *vs* Alcohol+Ex, *P* = 0.0004. **J** Peak value of each identified calcium transient. Nonparametric Kruskal-Wallis test statistic = 14.30, *P* = 0.0008. Dunn’s *post hoc* comparison, Saline *vs* Alcohol, *P* = 0.0284; Alcohol *vs* Alcohol+Ex, *P* = 0.0007. *n* = 159, 198, and 239 neurons from 4 mice in the Saline, Alcohol, and Alcohol+Ex group, respectively, in (**I**–**J**). **K** The matrix of heatmap showed the correlation of neuronal calcium activities from one specific field of view (FOV). **L** The comparison of averaged correlation efficiency. One-way ANOVA, *F*_(2, 30)_ = 5.157, *P* = 0.0114. Tukey’s *post-hoc* comparison, Saline *vs* Alcohol, *P*=0.0181; Alcohol *vs* Alcohol+Ex, *P* = 0.0293. *n* = 11 FOVs from 4 mice in each group. ns, no significant difference; *P < 0.05; **P < 0.01; ***P < 0.001; ****P < 0.0001. All data were presented as mean±SEM.

Motor learning ability is highly dependent on the dynamic change of neuronal spines in M1, we thus investigated the *de novo* spine turnover by performing alcohol administration and treadmill exercise in Thy1-YFP transgenic mice (Fig. 2A). Using transcranial 2-photon imaging of M1 apical spines before and after 3-day rotarod learning (Fig. 2B), we found the decreased spine density in alcohol group, while exercise training restored normal spine numbers (Fig. 2C). By analyzing the spine dynamics, alcohol expose decreased both formation and elimination of apical spines, and exercise training mainly elevated the spine formation rate (Fig. 2D–E), resulting in the higher overall turnover rate of the synapse (Fig. 2F). Such modulation of structural plasticity may form the substrate of improved motor learning functions under exercise scheme.

We next explored the functional relevance of these structural changes using *in vivo* calcium imaging of M1. By expressing genetically coded calcium sensor GCaMP6s into layer V of M1 using adeno-associated virus (AAV), the neuronal calcium activity was obtained using 2-photon imaging (Fig. 2G–H). Quantitative analysis found the decreased total calcium activities under alcohol exposure, and the effectively restored neuronal activity by exercise training (Fig. 2I). Further analysis showed a similar change of peak values of each calcium transient, which was depressed by alcohol and increased by exercise (Fig. 2J). Interestingly, although the total calcium transients were decreased, the alcohol-treated group presented a higher degree of synchronization among M1 neurons, which were decoupled by exercise training (Fig. 2K–L). This structural and functional evidence of cortical neurons showed the recovery of cortical functions by exercise training, possibly contributing to improved motor learning.

### Astrocytic Calcium Activity is Modulated by Exercise Training to Affect Motor Learning

**Fig. 3:**
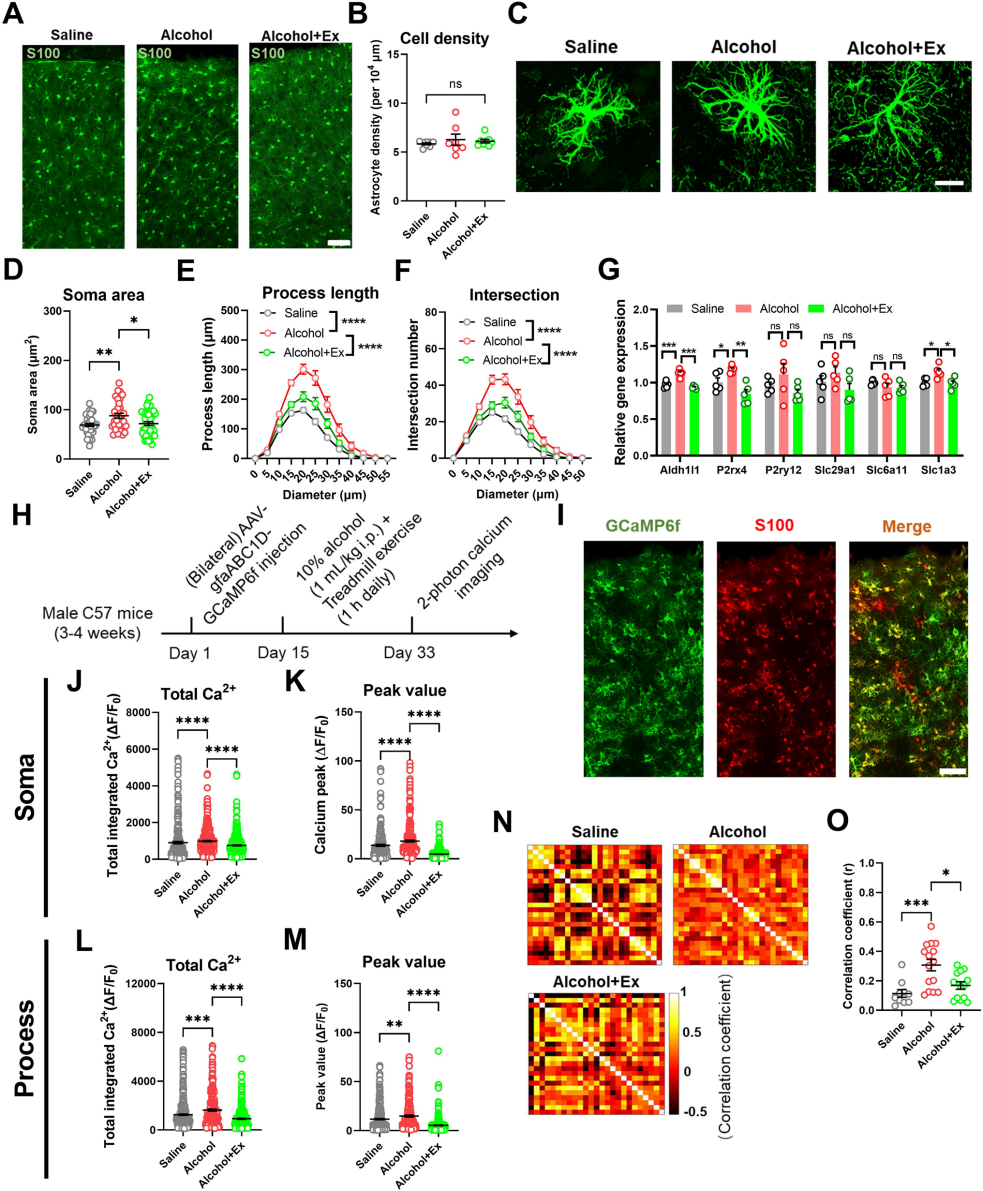
Exercise relieves the hyperreactivity of cortical astrocytes. **A** Representative immunofluorescent images showing the distribution of astrocytes (S100+) in M1. Scale bar, 100 μm. **B** The density of S100+ astrocytes in M1. One-way ANOVA, *F*_(2, 20)_ = 0.4523, *P* = 0.6425. *n* = 8, 7, and 8 mice in the Saline, Alcohol, and Alcohol+Ex groups, respectively. **C** Fluorescent images of individual astrocytes. Scale bar, 10 μm. **D** The somatic area of astrocytes. One-way ANOVA, *F*_(2, 113)_ = 6.069, *P* = 0.0031. Tukey’s *post-hoc* comparison, Saline *vs* Alcohol, *P* = 0.0041; Alcohol *vs* Alcohol+Ex, *P* = 0.0153. **E** The process length of the astrocyte within each radius range. Two-way ANOVA for the group factors, *F*_(2, 888)_ = 104.9, *P* < 0.0001. **F** The intersection number of the process branch. Two-way ANOVA for the group factors, *F*_(2, 888)_ = 85.70, *P* < 0.0001. *n* = 40, 33, and 43 cells extracted from 5 mice in the Saline, Alcohol, and Alcohol+Ex group, respectively, in (**D**–**F**). **G** The relative expression of genes related to astrocyte function. Multiple *t*-tests were used for comparisons between 2 specific groups. Alcohol *vs* Saline: *P* = 0.0008 for *Aldh1l1*, 0.0351 for *P2rx4*, 0.3707 for *P2ry12*, 0.3079 for *Slc29a1*, 0.3216 for *Slc6a11*, and 0.0145 for *Slc1a3*; Alcohol+Ex *vs* Alcohol: *P* = 0.0003 for *Aldh1l1*, 0.0033 for *P2rx4*, 0.1621 for *P2ry12*, 0.0796 for *Slc29a1*, 0.9052 for *Slc6a11*, and 0.0141 for *Slc1a3*. *n* = 5 mice per group. **H** Experimental scheme of astrocyte calcium imaging. **I** Representative images showing the expression of GCaMP6s in astrocytes within M1. Scale bar, 100 μm. **J** Total integrated calcium strength of astrocytic soma. Nonparametric Kruskal-Wallis test statistic = 45.20, *P* < 0.0001. Dunn’s *post-hoc* comparison, Saline *vs* Alcohol, *P* < 0.0001; Alcohol *vs* Alcohol+Ex, *P* < 0.0001. **K** Peak value of each identified calcium transient. Nonparametric Kruskal-Wallis test statistic = 331.9, *P* < 0.0001. Dunn’s *post hoc* comparison, Saline *vs* Alcohol, *P* < 0.0001; Alcohol *vs* Alcohol+Ex, *P* < 0.0001. *n* = 293, 389, and 444 soma from 4 mice in the Saline, Alcohol, and Alcohol+Ex group, respectively, in (**J**–**K**). **L** Total integrated calcium strength of the astrocytic process. Nonparametric Kruskal-Wallis test statistic = 52.26, *P* < 0.0001. Dunn’s *post-hoc* comparison, Saline *vs* Alcohol, *P* = 0.0005; Alcohol *vs* Alcohol+Ex, *P* < 0.0001. **M** Peak value of each identified calcium transient. Nonparametric Kruskal-Wallis test statistic = 153.6, *P* < 0.0001. Dunn’s *post-hoc* comparison, Saline *vs* Alcohol, *P* = 0.0046; Alcohol *vs* Alcohol+Ex, *P* < 0.0001. *n* = 364, 245, and 301 processes in the Saline, Alcohol, and Alcohol+Ex group, respectively, in (**L**–**M**). **N** The matrix of the heatmap showed the correlation of astrocytic calcium activities from one specific FOV. **O** The comparison of averaged correlation efficient. One-way ANOVA, *F*_(2, 30)_ = 5.157, *P* = 0.0114. Tukey’s *post-hoc* comparison, Saline *vs* Alcohol, *P* = 0.0008; Alcohol *vs* Alcohol+Ex, *P* = 0.0101. *n* = 10, 15, and 13 FOVs from 4 mice in the Saline, Alcohol, and Alcohol+Ex groups, respectively. ns, no significant difference; *P < 0.05; **P < 0.01; ***P < 0.001; ****P < 0.0001. All data were presented as mean±SEM.

When further analyzing the cellular mechanism of dysregulated cortical function, it is noticed that astrocytes play a crucial role in modulating synaptic plasticity [20] and neuronal activity [21]. We thus compared the M1 astrocyte density among three groups and found no change by either alcohol or exercise (Fig. 3A–B). However, further morphometric analysis showed distinct patterns of astrocyte processes (Fig. 3C). In particular, alcohol exposure enlarged the somatic area of astrocytes, and exercise training normalized soma size (Fig. 3D). The Sholl analysis of cellular branches showed increased process length by alcohol, and exercise training partially relieved such overgrowth (Fig. 3E). Similar patterns occurred for the number of processes, which can be evaluated by the interaction against the arbitrary concentric circles (Fig. 3F). These structural changes implied the reactive status of astrocytes by alcohol and exercise-mediated recovery. This can be supported by a series of molecular analyses, in which astrocytes marker gene (*Aldh1l*), adenosine receptor (*P2rx4*), and glutamate transporter (*Slc1a3*) showed elevated expression by alcohol, plus dampening levels after exercise training (Fig. 3G).

The morphological switch of astrocytes implied functional alternations, which were subsequently studied by *in vivo* calcium imaging in M1. Using similar strategies as those of cortical neurons, GCaMP6f was expressed by an astrocytic-specific promoter (gfaABC1D) carried by an AAV vehicle (Fig. 3H). The specificity of the calcium sensor was verified by immunolabelling with S100 (Fig. 3I). Quantitative analysis showed increased total calcium activities in both soma and process of M1 astrocytes by alcohol treatment, and the recovery toward normal levels in exercised animals (Fig. 3J, L). Such changes were mainly attributed to the modulation of calcium transient peaks (Fig. 3K, M). Furthermore, when we analyzed the correlation coefficient among astrocytes in each field of view, results showed increased synchronization of astrocytic calcium transients under alcohol, and decoupling by exercise training (Fig. 3N–O). These data collectively showed the hyper-reactivation of astrocytes by alcohol and functional recovery by treadmill exercise.

**Fig. 4:**
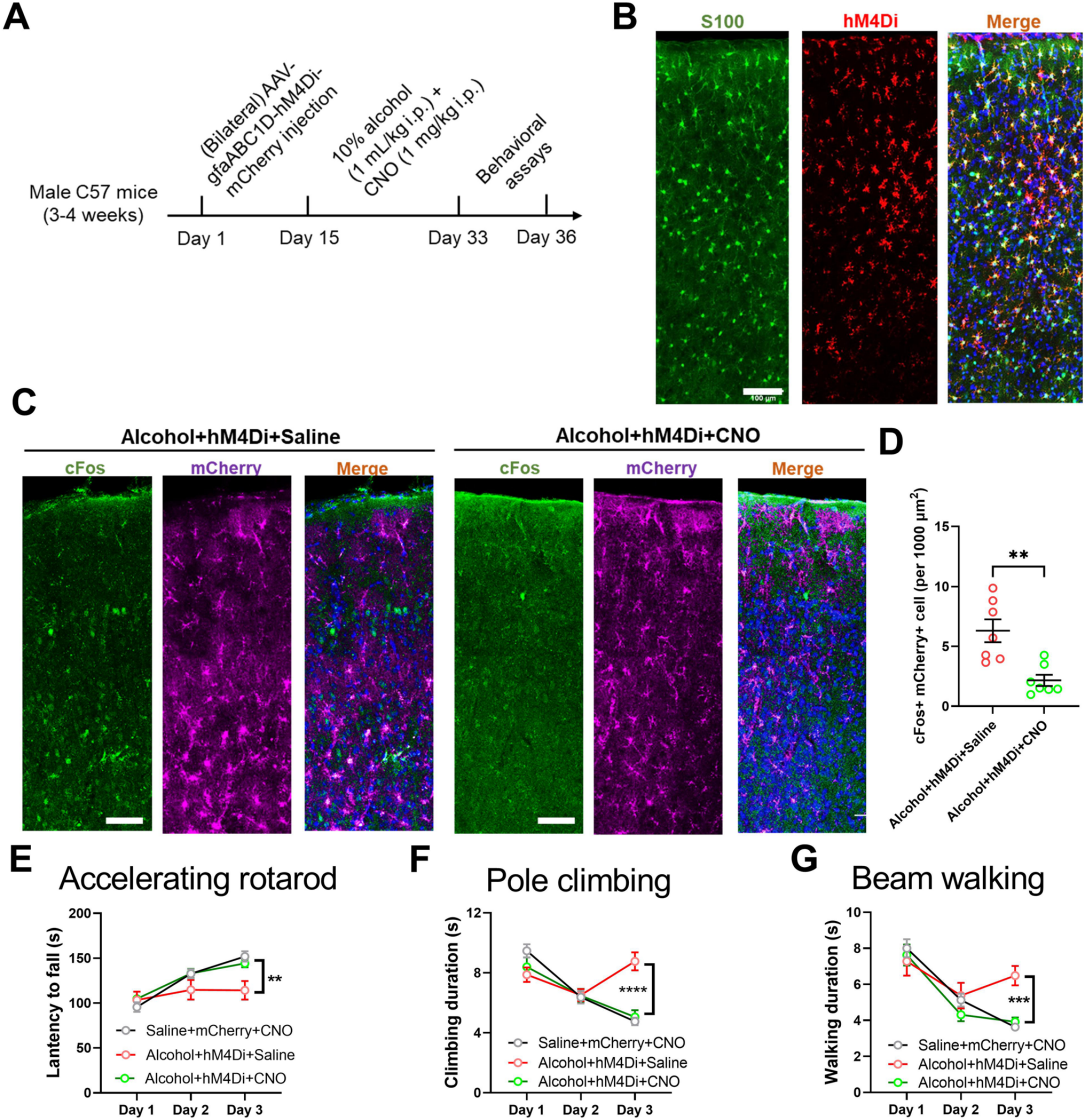
Chemogenetic inhibition of astrocytes relieves alcohol-induced motor deficits. **A** Experimental scheme of chemogenetic approaches. **B** Representative images showing the expression of hM4Di onto astrocytes. Scale bar, 100 μm. **C** Immunofluorescent images of cFos expression in M1 astrocytes. Scale bar, 100 μm. **D** Quantification of (**C**). Two-sample unpaired *t*-test, *t*_(12)_ = 3.904, *P* = 0.0021. *n* = 7 mice from each group. **E** Latency to fall during the 3-day accelerating rotarod assay. Two-way ANOVA for the group factor, *F*_(2, 27)_ = 5.233, *P* = 0.0012. **F** Time spent climbing the vertical pole. Two-way ANOVA for the group factor, *F*_(2, 27)_ = 13.50, *P* < 0.0001. **G** Duration in accomplishing the horizontal walking beam. Two-way ANOVA for the group factor, *F*_(2, 27)_ = 5.675, *P* = 0.0006. *n* = 8, 12, and 10 mice in Saline+mCherry+CNO, Alcohol+hM4D+Saline, Alcohol+hM4D+CNO group, respectively, in (**E**–**G**). **P < 0.01; ***P < 0.001; ****P < 0.0001. All data were presented as mean±SEM.

Lastly, we investigated if astrocytic activity was related to motor learning functions. Based on observed higher astrocytic calcium strength under alcohol exposure, we selectively expressed the inhibitory chemogenetic receptor hM4Di into M1 astrocytes (Fig. 4A). The specificity of chemogenetic receptor expression in astrocytes has been verified (Fig. 4B). With the introduction of specific ligand, clozapine N-oxide (CNO), we observed the suppression of astrocytic activity as shown by the expression of immediate early gene cFos (Fig. 4C–D). Moreover, we found an improvement in motor learning behaviors across the test battery (Fig. 4E–G), replicating the exercise effects. These data converged to show the modulation of both neuronal and astrocytic homeostasis by exercise training, to improve motor learning deficits induced by chronic alcohol exposure.

## Discussion

Astrocyte is the primary cell type for alcohol metabolism within the brain [6] and is subjected to damage caused by alcohol abuse [2]. The potential relationship between astrocytes and spine dynamics under alcohol exposure, however, remains unclear. Here we presented *in vivo* imaging data showing the deformation of M1 apical spines, in association with elevated astrocytic calcium activity after chronic alcohol intake. Moreover, exercise training was found to relieve the hyperreactivity of astrocytes, and to restore normal spine formation. These morphological changes ultimately contribute to improved motor learning behaviors, illustrating possible mechanisms of exercise in alleviating motor deficits caused by alcohol abuse.

Deficits in complex motor learning are one common symptom in alcohol abuse individuals. Its pathological mechanisms can be attributed to both peripheral and central factors. In skeletal muscles, alcohol exposure led to dysregulated muscle metabolism [22] and myopathy diseases [23], resulting in impaired motor functions. Alcohol may also disrupt neuromuscular junctions to affect voluntary movement [24]. In our data, however, this peripheral model can be rejected as unchanged grip strength (Fig. 1D) implied intact neuromuscular connections. In the brain, alcohol has been recognized to affect the neural network of the cerebellum, which is critical for motor coordination. For example, the synaptic plasticity of Purkinje neurons is modulated by alcohol exposure [25]. In addition to cerebellar nuclei, voluntary motor control involves a complex neural network including brain stem nuclei, striatum, thalamus, and cortical regions. Among these brain areas, M1 is recognized as the higher center for the acquisition of complex motor skills, as supported by high dynamics of synaptic structural turnover [26, 27]. Our previous work has also revealed the critical role of synaptogenesis of M1 in motor skill learning under both normal [19] and cocaine-exposed conditions [12]. Therefore, in our work, the motor cortex was mainly investigated, and results revealed the involvement of M1 in alcohol-dependent motor disorders. The current data, however, does not preclude the participation of other brain nuclei in the exercise-modified neural network adaption to improve alcohol-induced motor deficits, as current knowledge has suggested the neuroprotective effect of the cerebellum [28] and striatum [29].

Astrocytic function is closely related to neuronal activity and synaptic plasticity. Previous works have shown the critical role of astrocyte-neuron crosstalk in synaptogenesis either during early developmental stages [16] or in adult brains [30]. From a functional perspective, astrocyte cooperates with neurons to mediate long-term neural plasticity [31], thus contributing to learning and memory functions. In our work, *in vivo* imaging data of apical spines and astrocytic calcium activity supports the interplay between astrocytes and synaptogenesis. Recent studies are gradually revealing the molecular mechanisms for astrocyte-mediated spine formation, including extracellular vesicles [32], clusterin [33], complement factors [34], and neurotrophic factors [35]. Although the current study did not analyze the molecular mediator of exercise-mediated astrocytic-neuron crosstalk, our previous works covering exercise-related neurotrophic factors [9] may provide candidates for further exploration.

In the current work, alcohol abuse depressed neuronal calcium activity but elevated astrocytic calcium strengths, and treadmill exercise reversed such pathological changes. Current knowledge agreed with our findings, by showing elevated astrocytic activity *in vitro* in response to acute alcohol exposure [36]. Moreover, another study also found elevated activity of Ca^2+^/CaM-dependent protein kinase in primary cultured cortical astrocytes under alcohol treatment [37], supporting the elevated calcium activity in our observation. When studying the relationship between astrocytic and neuronal calcium activities, the activation of cortical neurons might imply higher astrocytic activity, as shown by previous studies in which neuron and astrocyte activity were spatially and temporally coordinated [38]. However, under our experimental scheme, we observed decreased calcium activity of astrocytes by treadmill exercise, probably reflecting the relief of cellular hyper-reactivity by alcohol exposure.

How physical exercise affects astrocytic morphology and activity is one interesting issue. A region-specific study reported unchanged astrocyte numbers in PFC under exercise, plus decreased complexity of cellular processes after 4 weeks of training [39]. In another study using a retinal degeneration model, treadmill exercise promotes the arborization of the astrocytic process [40]. These seemingly contradictory data revealed the critical role of exercise in maintaining the homeostasis of astrocytes. In our work, alcohol infusion induced the overgrowth of the astrocytic process, as agreed with previous reports under 3-week alcohol exposure [41]. It is thus proposed that exercise training may help to relieve astrocyte overgrowth under pathological conditions, just as those reported in Alzheimer’s disease [42] and cerebral hypoperfusion [43]. When investigating the detailed molecular mechanisms, available studies mainly focused on functional proteins within astrocytes such as aquaporin 4 [44]. In the future, the metabolic switch carried by exercise should be studied as exercise-related metabolites such as lactate may affect astrocyte morphology and function [45].

In this work, we adopted the intraperitoneal injection model of alcohol, which is one widely adopted approach in mouse studies. The major advantage of this model is the relatively constant alcohol intake in each animal, as rodents present highly variable alcohol preferences using the 2-bottle voluntary intake model [46]. Besides studying brain function, this intraperitoneal model has been reported to affect bone metabolism [47], lipid oxidation [48], or energy homeostasis [49]. More importantly, the administration of alcohol profoundly affects the hepatic function, which further mediates the brain microenvironment *via* the metabolic byproducts [50] or inflammatory response [51]. Since exercise has been reported to improve hepatic function under alcohol abuse [52], it is possible that exercise training reshapes peripheral homeostasis, contributing to the astrocytic remodeling in the cortex.

The current work has certain limitations and weaknesses. Here we only include male mice but not female ones. Current knowledge has suggested sex-specific traits of brain adaptions to alcohol exposure [53]. For example, females are more sensitive to reinforcing alcohol self-administration [54], and their hippocampal neurogenesis is more vulnerable than males in a rodent study [55]. In terms of glial cells, female individuals are more sensitive to alcohol toxicity. In a rat study that combined both binge alcohol and physical exercise, females presented increased microglial number and neuroinflammatory response than males in the hippocampus and mPFC [56]. In astrocytes, a sex-specific effect was also reported, as hippocampal astrocytes exhibited increased expression of inflammatory cytokines under alcohol exposure only in females [57]. These lines of evidence imply the possibly better effects of exercise training on female alcohol-takers, and future works can be pursued to examine the sex-specific effect under the treadmill scheme.

In sum, our work collectively suggested the disruption of neuronal and astrocytic activity, plus loss of dendritic spines under chronic alcohol exposure, leading to motor learning deficits. Treadmill exercise, on the other hand, effectively restored cellular homeostasis *via* inhibiting astrocyte reactivity and improving synaptic structure and function, contributing to the recovery of motor learning. Our data shed more insights into the neuron-astrocyte crosstalk during exercise intervention of alcohol abuse.

### Supplementary Information

Below is the link to the electronic supplementary material.

Supplementary file1 (PDF 279 KB)
