# Supplementary material for: Treadmill Exercise Reshapes Cortical Astrocytic and Neuronal Activity to Improve Motor Learning Deficits Under Chronic Alcohol Exposure
Source: Neurosci Bull. 2024 May 28;40(9):1287–98. doi: 10.1007/s12264-024-01226-x (PMC11365901; doi:10.1007/s12264-024-01226-x)
Supplement: Supplementary file 1 — Supplementary file1 (PDF 279 KB) [file 12264_2024_1226_MOESM1_ESM.pdf]

Supplemental Materials

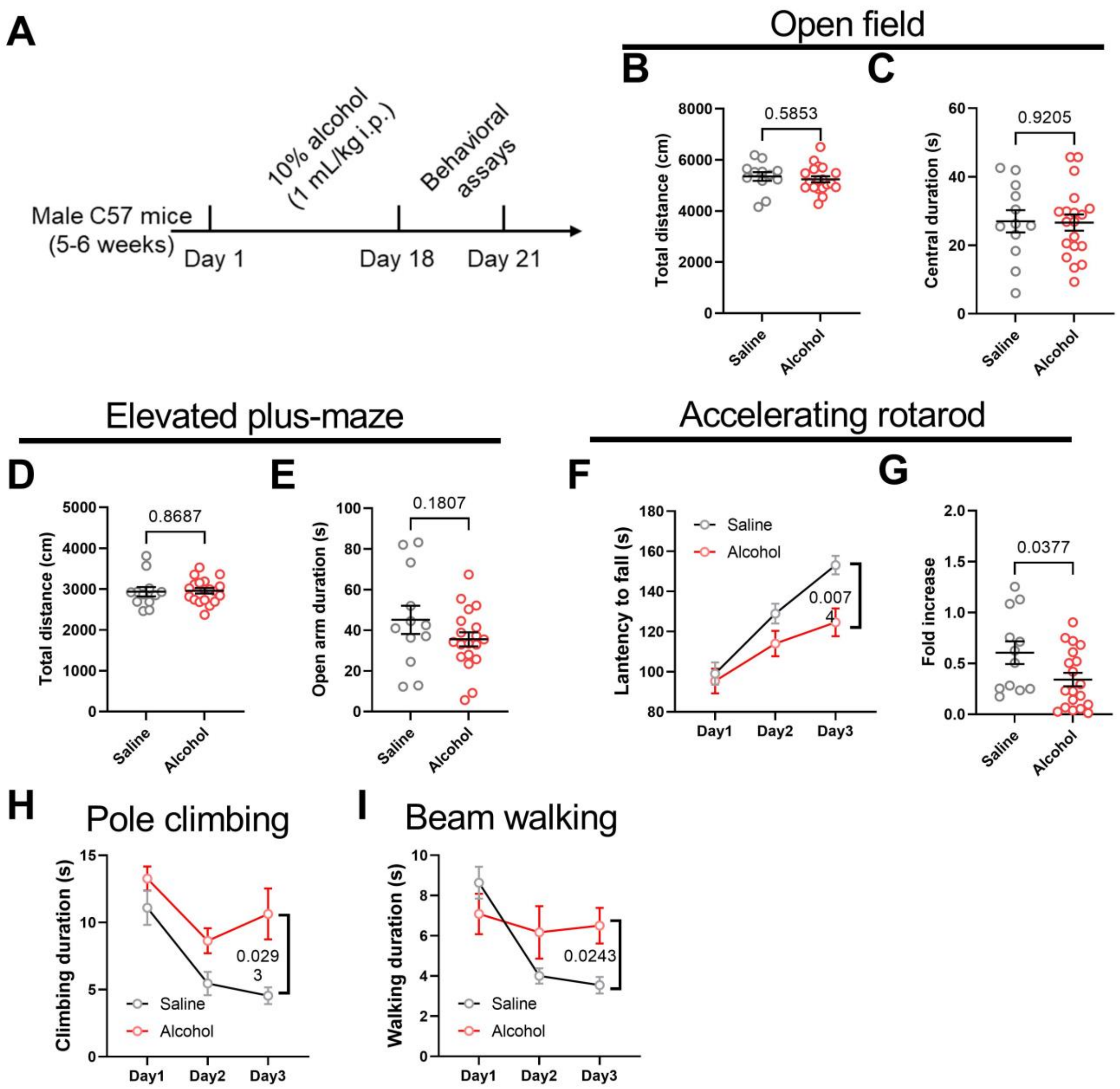

Fig. S1 Chronic alcohol infusion induced motor learning deficits. **A** Experimental scheme of alcohol infusion. **B** Total distance in the open field. Two-sample *t*-test, $t_{(29)} = 0.5518$, $P = 0.5853$. **C** Central duration in the open field. Two-sample *t*-test, $t_{(29)} = 0.1007$, $P = 0.9205$. **D** Total distance traveled in the elevated plus-maze. Two-sample *t*-test, $t_{(29)} = 0.1668$, $P = 0.8687$. **E** Time spent in the open arm. Two-sample *t*-test, $t_{(29)} = 1.372$, $P = 0.1807$. **F** Latency to fall during the 3-day accelerating rotarod

assay. Two-way ANOVA for the group factor, $F_{(1, 29)} = 5.836$, $P = 0.0049$. **G** The improvement of rotarod latency. Two-sample *t*-test, $t_{(29)} = 2.178$, $P = 0.0377$. **H** Time spent climbing the vertical pole. Two-way ANOVA for the group factor, $F_{(1, 29)} = 9.423$, $P = 0.0060$. **I** Duration in accomplishing the horizontal walking beam. Two-way ANOVA for the group factor, $F_{(1, 29)} = 4.759$, $P = 0.0137$. $n = 12$ and 19 mice in the Saline and Alcohol group, respectively. Tukey's multiple comparison test was used to make the comparison between 2 specific groups. In (**F, H–I**), *P* values of the comparison between the Alcohol and Alcohol+Ex groups were annotated. All data were presented as mean±SEM.

Table S1 Full list of viral vectors and antibodies used.

(A) Viral vectors

Virus	*Source*	*Identifier*	*Titer*
rAAV2/9-CaMKII-GCaMP6s-WPRE-pA	BrainVTA, Wuhan	PT-0110	2×10^{12} vg/mL
AAV2/5-GfaABC1D-mCherry-WPRE-SV40-pA	BrainVTA, Wuhan	PT-1175	5×10^{11} vg/mL
AAV2/5-GfaABC1D-GCaMp6f-WPRE-pA	BrainVTA, Wuhan	PT-2560	5×10^{11} vg/mL
rAAV2/5-GfaABC1D-hM4D-mCherry-WPRE-SV40-pA	BrainVTA, Wuhan	PT-1439	5×10^{11} vg/mL

(B) Antibodies

Antibodies	*Source*	*Identifier*	*Diluent*
S100-beta antibody primary antibodies	Sigma-Aldrich	SAB5600115	1:500
Glial Fibrillary Acidic Protein (GFAP) primary antibodies	Abcam, USA	ab4674	1:500
cFos primary antibodies	Synaptic Systems, DE	226-308	1:1000
Donkey anti-rabbit Alexa Fluor 594	Jackson ImmunoResearch, UK	711-585-152	1:500
Goat anti-rabbit Alexa Fluor 488	Invitrogen, USA	A11008	1:500
Goat anti-chicken Alexa Fluor 488	Invitrogen, USA	A11039	1:500
Goat anti-guinea pig Alexa Fluor 647	Invitrogen, USA	A21450	1:500
